# Impact of Tree Pollen Distribution on Allergic Diseases in Serbia: Evidence of Implementation of Allergen Immunotherapy to *Betula verrucosa*

**DOI:** 10.3390/medicina56020059

**Published:** 2020-02-04

**Authors:** Rajna Minić, Mirjana Josipović, Vesna Tomić Spirić, Marija Gavrović-Jankulović, Aleksandra Perić Popadić, Ivana Prokopijević, Ana Ljubičić, Danijela Stamenković, Lidija Burazer

**Affiliations:** 1 Institute of Virology, Vaccines and Sera, “Torlak”, Vojvode Stepe 458, 11000 Belgrade, Serbialburazer@torlak.rs (L.B.); 2 Environmental Protection Agency, Ruže Jovanović 27a, 11060 Beograd, Serbia; 3 Faculty of Medicine, University of Belgrade, Doktora Subotica 8, 11000 Belgrade, Serbia; 4 Clinic for Allergology and Immunology, Clinical Centre of Serbia, Koste Todorovića 2, 11000 Belgrade, Serbia; 5 Faculty of Chemistry, University of Belgrade, Studentski trg 16, 11000 Belgrade, Serbia

**Keywords:** aerobiology, *Betula verrucosa*, pollination, allergic disorders, allergen immunotherapy

## Abstract

*Background and objectives:* The relationship between air pollen quantity and the sensitization of allergic patients is crucial for both the diagnosis and treatment of allergic diseases. Weather conditions influence the distribution of allergenic pollen and increases in pollen concentration may negatively affect the health of allergic patients. The aim of this study was to analyze the implementation of allergen immunotherapy with regard to air pollen concentration. *Material and Methods*: Here we examined the relationship between *Betula* air pollen concentration and the usage of *Betula verrucosa* allergen immunotherapy in Serbia. Examination covered the period from 2015 to 2018. Measurement of airborne pollen concentration was performed with Lanzoni volumetric pollen traps. The evidence of the usage of sublingual allergen immunotherapy (SLIT) was gathered from patients with documented sensitization to specific pollen. *Results*: During this period tree pollens were represented with 58% ± 21% of all measured air pollen species, while *Betula* pollen represented 15% ± 8% of all tree pollens. *Betula* pollination peaked in April. Allergen immunotherapy to *Betula verrucosa* in Serbia is entirely conducted as sublingual immunotherapy and represents 47.1% ± 1.4% of issued tree pollen SLIT. The use of pollen SLIT increased by 68% from 2015 to 2018, with an even greater increase in usage recorded for *Betula* SLIT—80%. *Conclusions:* This analysis shows a clear causative relationship between pollination and the type/prevalence of applied allergen immunotherapy. Information about the flowering seasons of allergenic plants is very important for people who suffer from allergy, for clinical allergologists, as well as for governing authorities. The presented data is of practical importance to the proper timing of immunotherapy initiation and of importance for urban landscaping. The obtained data can be the starting point for the instatement of a thorough epidemiological study and the inclusion of Serbia on the pollen map of Europe.

## 1. Introduction

The prevalence of allergic diseases has increased into a pandemic health problem, with estimates being that half a billion people worldwide suffer from allergic rhinitis [1]. Allergic diseases are an increasing health problem in today’s world including Europe [2,3,4,5]. Different kinds of air suspended allergens can cause allergic diseases, which are manifested as seasonal and/or perennial conjunctivitis, rhinitis, or asthma. Pollen allergy has a common clinical impact across Europe and impairs patients’ quality of life. The classification of pollen-induced allergies is based on the source of allergens: grass, trees, or weed. Exposure to a particular type of pollen is a starting point and a very significant initial factor for the development of allergic diseases. Wind pollination which is found in 10%–18% of all flowering plants is considered the main cause of pollen sensitization [6]. Pollen concentration, the period of pollination, as well as pollen allergenicity all depend on climate conditions [2,7,8]. Serbia’s geographical position is such that it is characterized by moderate continental climate, with more or less pronounced local characteristics [9]. In accordance with the climate conditions in Serbia pollen from trees, grasses, and weeds is present in the air in significant amounts. The period of tree pollination lasts from February to May. The measurement of air pollen concentration is important for monitoring allergenic plant distribution and yearly variations [10]. The importance of allergen pollen measurement is reflected in the timely application of allergen immunotherapy. In 2002 the Environmental Protection Agency, member of the European Aeroallergen Network started with pollen monitoring in one station. Measurement of pollen air concentration in Serbia has since progressed so that it currently includes over 19 measuring stations. Allergen immunotherapy (AIT) is an effective treatment for allergic respiratory diseases in children and adults. In current clinical practice, immunotherapy is delivered either subcutaneously (SCIT) or sublingually (SLIT). The efficacy of both modalities of AIT has been confirmed by numerous randomized controlled trials and meta-analyses [11,12,13,14,15,16]. Allergen immunotherapy formulations can be produced as crude extracts, chemically modified allergens, allergoids, or recombinant allergen immunotherapy. In this study sublingual immunotherapy with crude allergen extracts was analyzed. In order to estimate tree pollen allergy prevalence in Serbia and the dominant allergenic tree species, an analysis of issued AIT was performed for the time period of four years (2015–2018). We focused on the analysis of measured tree pollen species in the air and the correlation with the implementation of allergen immunotherapy in order to obtain information on the causal relationship. Due to the fact that *Betula verrucosa* AIT is almost exclusively SLIT only this kind of therapy was analyzed.

## 2. Materials and Methods

### 2.1. Pollen Monitoring in Serbia

Measurement of airborne pollen concentration was performed routinely through The Serbian Environmental Protection Agency, Sector for Quality Control and State of the Environment, Department for Air Quality Control, Section for Air Quality Monitoring, Group for Monitoring of Allergen Pollen. Lanzoni volumetric pollen traps, utilizing the volumetric method [17] were used to monitor pollen concentrations. The units of expression are number of pollen grains/m^3^ air. Pollen grain count included among trees, representative species from the following families: *Moraceae*, *Pinaceae*, *Cypress/Taxa.*, *Ulmaceae*, and genera *Acer*, *Aesculus*, *Alnus*, *Betula*, *Carpinus*, *Corylus*, *Fagus*, *Fraxinus*, *Juglans*, *Platanus*, *Populus*, *Quercus*, *Salix*, *Tilia*. For graphical presentation daily values were averaged in order to obtain average weekly values and weeks are taken as the smallest unit of measurement. In order to compare “absolute quantities” of air pollen, daily values were used to obtain average weekly pollen values which were summed to obtain yearly values. During the time period analyzed, 19 measuring stations were operable, and 15 stations were included in the analysis. 

### 2.2. Evaluation of the Usage of Allergen Immunotherapy

SLIT is issued by the local producers (Institute of Virology, Vaccines and Sera “Torlak”) according to strict guidelines in the form of “name prepared product” to patients with original documentation of allergen sensitization evidenced by a prescription from the allergist. Allergen extracts were prepared according to strictly defined protocols from raw materials (pollen grains) by defatting and phosphate buffer extraction for 48 h at 2–8 °C, followed by sterile filtration [18]. Data on SLIT demand was extracted for the four successive years 2015—2018. From the collected data information was obtained about the newly initiated therapy for each of the analyzed years as well as of the ongoing therapy consumption, as the therapy lasts several years. Tree pollen is a mixture consisting of selected species from the orders Fagales, Laminales, Proteales, and Pinales (*Abies alba, Alnus glutinosa*, *Betula verrucosa*, *Carpinus betulus*, *Corylus avelana*, *Fraxinus nigra*, *Juglans regia*, *Platanus* spp., *Populus canadensis*, *Populus nigra, Querques* spp., *Salix alba*, *Sambucus nigra, Tilia* spp., *Ulmus* spp., *Pinus nigra*).

### 2.3. Statistical Analysis

All statistical analyses were performed with GraphPad Prism software. Normality of the data was tested by the Shapiro–Wilk test. For comparisons the Friedman test was used, with Dunn’s multiple comparison test. The limit of statistical significance was considered *p* < 0.05.

## 3. Results

### 3.1. Pollen Monitoring Data 

**Figure 1:**
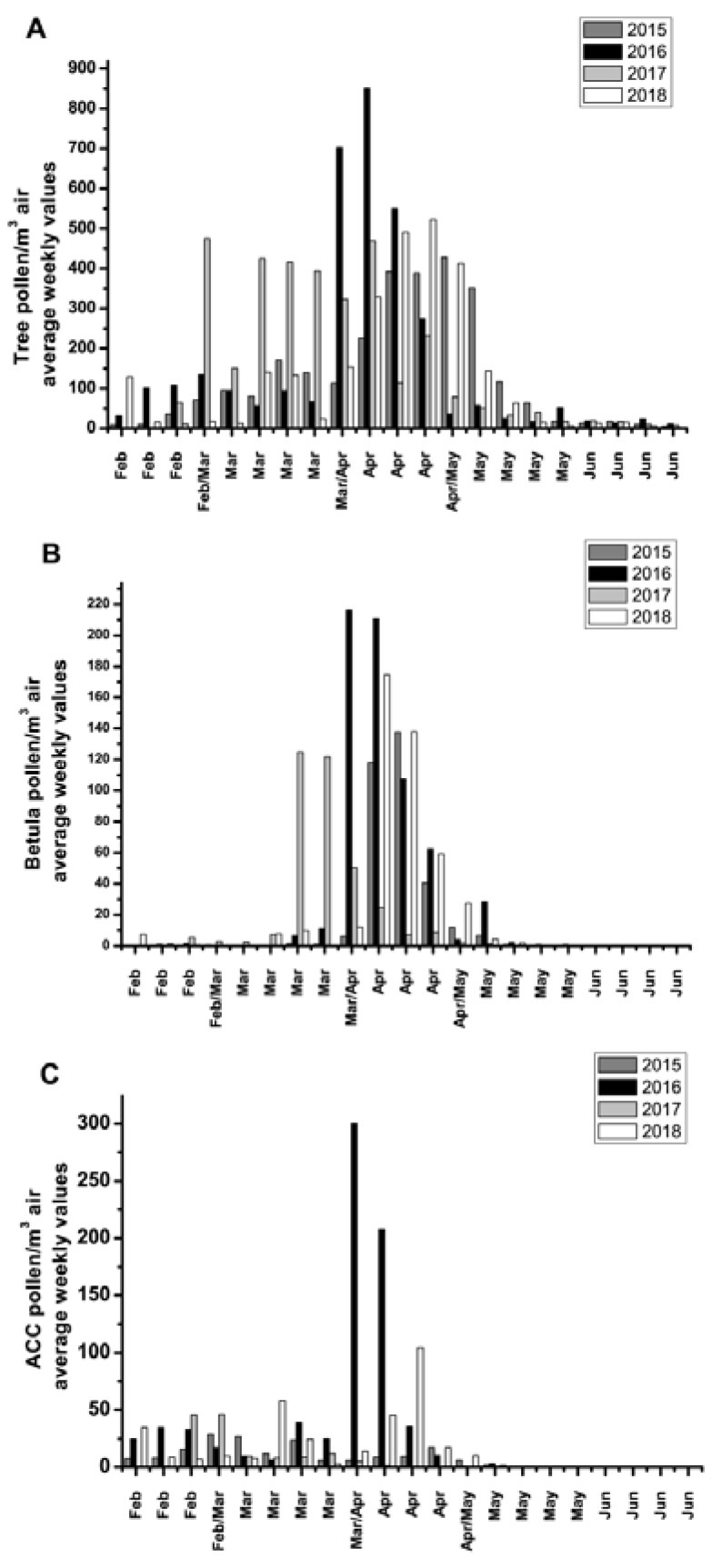
Yearly quantities and duration of increased air pollen concentrations; average values from the nineteen individual measuring stations from Serbia. (**A**) Total tree pollen, (**B**) *Betula* spp. pollen, (**C**) ACC (*Alnus, Corrylus* and *Carpinus* spp.) pollen.

The pollen monitoring season started in February each year. During the followed time period a relatively constant total tree pollen concentration was noted for particular measuring locations throughout Serbia, which weekly averages, generally, below 500 tree pollen grains per cubic meter of air, Figure 1A, except for the 2016 increase. A similar situation was observed for *Betula* spp. where the average weekly concentrations across Serbia were generally below 140 pollen grains per cubic meter air, except for the 2016 and 2018 increase, Figure 1B. On average *Betula* pollen was represented with 15% ± 8% of all tree pollen species detected during the measured time period 2015–2018.

Lower air concentrations (weekly averages) of summed pollen belonging to *Alnus, Corrylus,* and *Carpinus* species were noted in the measured time period, and were generally below 50 pollen grains/m^3^ air, again except for 2016 and a smaller increase in April 2018, Figure 1C. 

**Figure 2:**
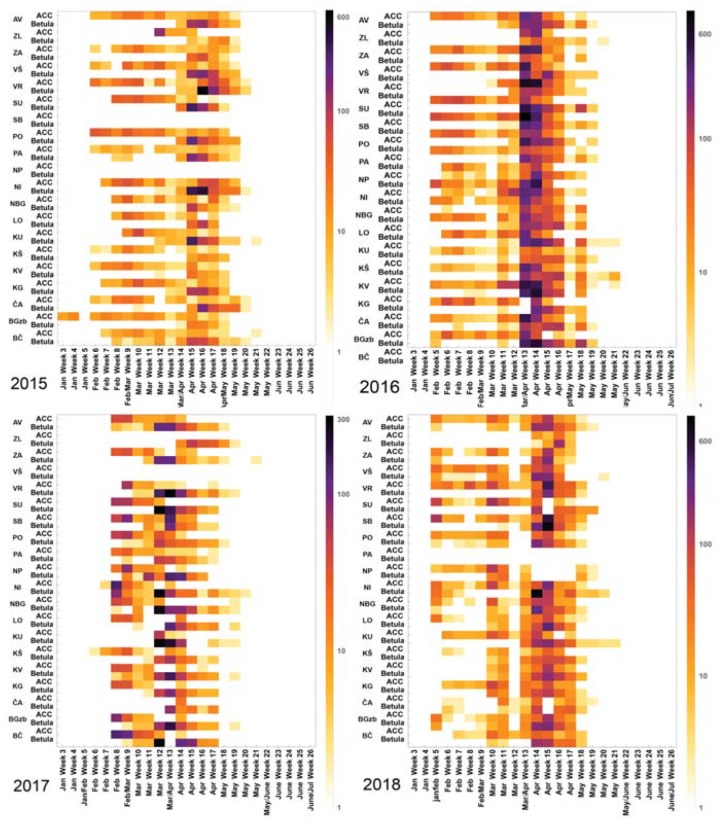
*Betula* spp. and ACC (*Alnus, Corrylus* and *Carpinus* spp.) pollen concentration timeline across 19 individual measuring stations in Serbia, shown as average weekly values. Time period 2015–2018. AV—Average values; Particular measuring locations: ZL—Zlatibor; ZA—Zaječar; VŠ—Vršac; VR—Vranje; SU—Subotica; SB—Sokobanja; PO—Požarevac; PA—Pančevo; NP—Novi Pazar; NI—Niš; NBG—Novi Beograd; LO—Loznica; KU—Kula; KŠ—Kruševac; KV—Kraljevo; KG—Kragujevac; ČA—Čačak; BGzb—Beograd, Zeleno brdo; BČ—Bečej.

The first recorded tree pollen species appearing in early spring belonged to *Corylus*, followed by *Alnus* (both Betulaceae) and *Cupress* (not shown), while *Betula* was detected first time in mid-March/early April, with peaks in early April, Figure 2. Average weekly pollen concentrations of *Betula* and *Alnus, Corrylus, Carpinus* are shown for each of the analyzed years and an increase in average pollen concentrations and the duration of pollination detected in 2016 is shown in Figure 2.

**Figure 3:**
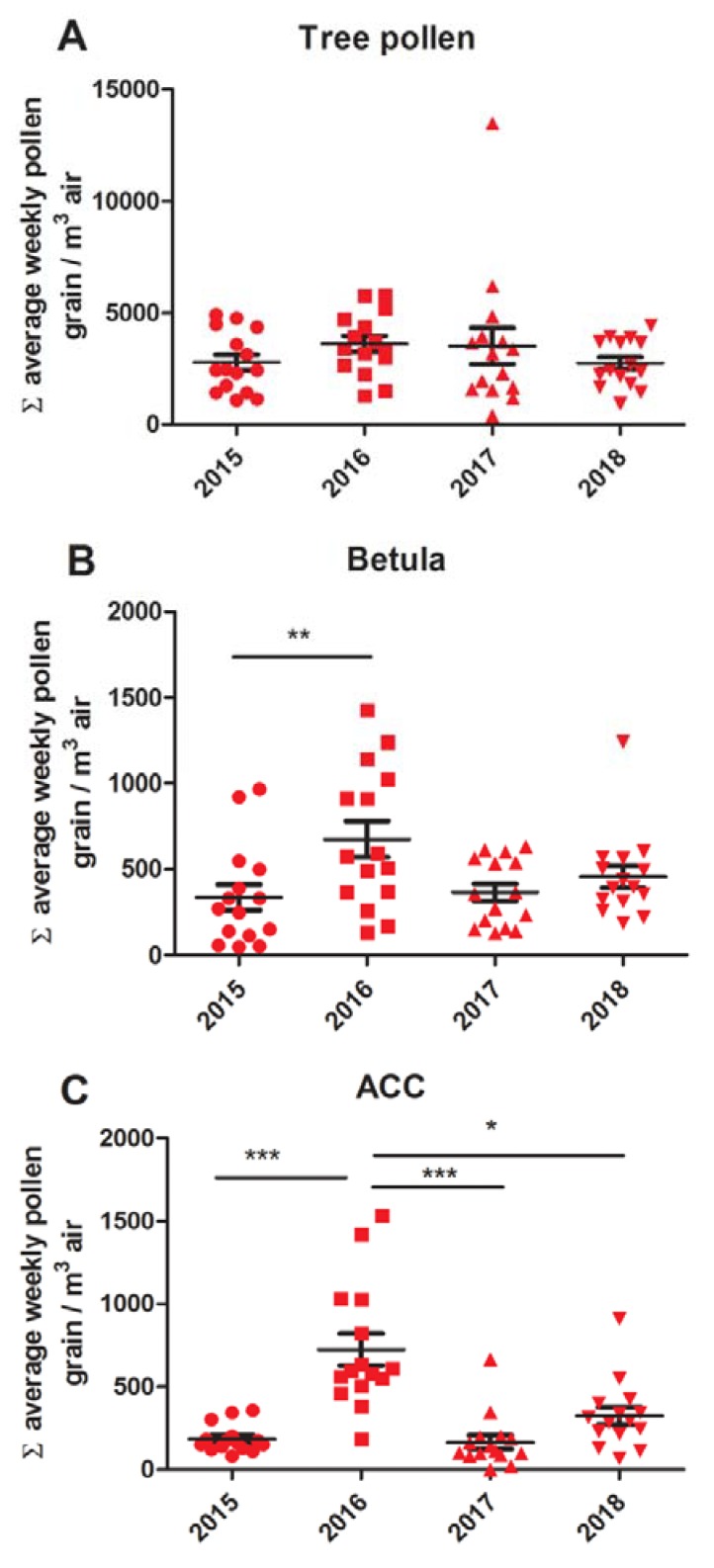
Statistical analysis of yearly variation in pollen air concentration viewed as the sum of average weekly values. (**A**) Tree pollen; (**B**) *Betula* spp. pollen; (**C**) *Alnus* + *Corylus* + *Carpinus* spp. pollen. Statistically significant differences are indicated as follows: * *p* < 0.05; ** *p* < 0.01; *** *p* < 0.001.

The air concentration of total tree pollen did not change significantly during the observed time period (Figure 3A), while the air concentration of *Betula* spp. pollen increased significantly in 2016 at all measuring locations Figure 3B.

A significant increase in the concentration of *Betula* pollen was observed in 2016, (Figure 3B), which was followed by the increase in other Betulaceae family members, *Corylus*, *Alnus,* and *Carpinus* (ACC), which were also significantly higher in 2016, Figure 3C.

### 3.2. Analysis on the Usage of Tree Pollen SLIT

The analysis of ongoing therapy consumption revealed that on average 19.0% ± 1.7% of issued sublingual immunotherapy included tree pollens during the three consecutive years chosen, Table 1. 

Most of the patients allergic to tree pollen were given *Betula verucossa* for the therapeutic treatment. This specific therapy is the most prevalent single allergen immunotherapy among trees, with a proportion of 47.1% ± 1.4% during the analyzed time period and with percentages increasing in 2016 and 2017, Table 2.

Regarding the usage of SLIT which included tree pollen, in the analyzed time period, the highest demand was for Mixtures including Tree pollen 30.7% ± 2.3% and *Betula* 47.1% ± 1.4%, whereas Tree pollen SLIT was present with 13.5% ± 0.6%. *Corylus* SLIT was represented with 3.4% ± 0.8% while other individual tree species were represented with less than 3%, on average, Table 2.

The increase in Betulaceae air pollen concentration influencing the application of allergen specific immunotherapy was especially pronounced in the analysis of yearly SLIT initiations. The consumption of *Betula* SLIT increased from 30% in 2015, to 38.4% in 2016. The absolute numbers are shown in Table 3. This increase was also noticed in 2017 and 2018 but to a lesser extent.

## 4. Discussion

As was recently concluded by Asam and coworkers, the prevalence of sensitization in a given population highly depends on the geographic location and thereafter on the pollen exposure pattern, hence tree pollen allergies emerge as a problem in industrialized countries within temperate climate zones [19]. Allergenic symptoms are related to pollen in a dose-dependent manner. Aerobiological and allergological measurements of the presence and prevalence of airborne pollens make it possible to design pollen calendars with the approximate flowering periods of the plants in the sampling area. The application of AIT for different pollen species begins in the pre-flowering season. The European Academy of Allergy and Clinical Immunology in a recently published position paper gave new definitions on pollen season and peak pollen period start and end, which are very important for the use of AIT [20]. Airborne pollen monitoring is therefore one of the key parameters for the timely implementation of AIT. Although allergen immunotherapy is still relatively frequently used as a mixture of different pollen species, there has been an increase in prescription of monovalent therapy. In Serbia *Betula* is one of the therapies that has mostly been issued as a monovalent therapy. Here we identified *Betula verrucosa* as the main allergenic tree species in Serbia, which was based on the analysis of therapy prescriptions gathered at the sole producer/distributor of allergen immunotherapy on the Serbian market. This is in accordance with the data from the region, as *Betula verrucosa* is, among trees, the most allergenic pollen producer in northern, central, and eastern Europe [5]. In addition, we noted how yearly fluctuations in pollen air concentration impact the allergic population, as evidenced by increased allergen specific therapy demand. In 2016, in the initial therapy treatment *Betula verucosa* leads with 38.4%, highlighting the increase in air pollen concentration which occurred that year immediately impacting patients.

In 2016 an increase in pollen concentration of other Betulaceae family members was also noted, which might have contributed to the increase of *Betula* therapy demand. Namely the analyzed Betulaceae family members were found to be homologous, as pre-absorption of 102 sera with a combination of recombinant rBet v 1 and rBet v 2, inhibited the binding of IgE to whole tree pollen extracts to a large extent (to hornbeam: 77%, to hazel: 80%, and to alder: 88%) [21]. *Betula* is by far the most allergenic Betulaceae family member and immunotherapy to these other species is rarely prescribed in Serbia. Pollen from other species of the Betulacea family does not appear to be such a potent allergen, possibly because of its lower abundance. Another reason might be that because of the high IgE cross reactivity between the species, where for instance *Alnus* sensitized patients react with *Betula* in diagnostic procedures, resulting in *Alnus* therapy being significantly less prescribed.

Pollen aerobiology can therefore be used to predict future allergen immunotherapy consumption dynamics.

Our findings correlate with the published data that the development of sustained sensitization is associated with exposure to a particular pollen species. Therefore, it is not surprising that *Betula* is the most prevalent allergenic tree pollen in Serbia, as it is also the most frequent cause of tree pollen allergy in Europe [5]. 

Various factors can contribute to the development of allergic diseases and it is meaningful to conduct thorough epidemiological analyses with the introduction of specific environmental parameters [22,23]. The lack of a comprehensive epidemiological study in Serbia makes monitoring difficult and a baseline for future studies is presented here.

Certainly, the measurement of pollen concentration in the relevant areas in Serbia (19 measuring locations covering the country) provides important information on pollen species including allergenic pollen. The data presented is not only of importance to allergen specific immunotherapy initiation timing but also of great importance for the policy of planting trees in city parks.

## 5. Conclusions

The increase in Betulaceae pollen air concentration in 2016 led to the increase in *Betula verrucosa* SLIT consumption the same year. It is known that different environmental factors contribute to the development of allergic diseases and it is very important to continue aerobiological analysis coupled with monitoring of a patient’s sensitivity to allergenic pollen, especially with the introduction of environmental parameters, like average temperatures, precipitation, air pollution and others. Big data analysis makes it possible to identify cause and effect relationships. The lack of a comprehensive epidemiological study in Serbia makes monitoring difficult but this study provides a baseline for future studies regarding tree pollen allergies in Serbia, which are important for public health and healthcare expenditure planning. In the future, trials of this type should be supplemented with data relating to the severity of patients’ allergic disease. 

## Figures and Tables

**Table 1:** Sublingual immunotherapy (SLIT) and SLIT initiations divided into Grass and weed therapy and Trees therapy issued to patients, shown as percentages of total issued SLIT.

	Total Yearly Therapy %		Total Initiations Therapy %	
Year	Grass and Weed	Tree	Grass and Weed	Tree
2015	83.1	17.0	80.9	19.1
2016	80.7	19.3	72.2	27.8
2017	78.8	21.2	78.6	21.4
2018	81.4	18.6	78.0	22.0

**Table 2:** Tree pollen sublingual immunotherapy issued to patients shown as percentages of total SLIT.

Total Yearly Therapy %								
Year	Mixtures Including Tree Pollen	Tree Pollen	*Betula*	*Corylus*	*Alnus*	*Juglans*	*Tilia*	*Quercus*
2015	28.2	13.4	46.1	4.4	0.7	3.0	4.0	0.2
2016	31.2	13.7	48.1	3.5	0.4	2.2	0.4	0.4
2017	29.7	12.7	48.4	3.1	0.5	3.1	2.0	0.5
2018	33.5	14.0	45.6	2.5	0.0	2.3	2.0	0.0

**Table 3:** Tree pollen sublingual immunotherapy initiations issued to patients.

Therapy Initiations %								
Year	Mixtures Including Tree Pollen	Tree Pollen	*Betula*	*Corylus*	*Alnus*	*Juglans*	*Tilia*	*Quercus*
2015	59	10	36	5	1	2	6	1
2016	71	38	73	4	1	2	0	1
2017	75	18	57	0	0	3	3	0
2018	78	26	53	4	0	4	3	0
